# The value of ^18^F-AlF-NOTATATE PET/CT in restaging high-risk neuroblastoma after chemotherapy

**DOI:** 10.3389/fmed.2025.1596272

**Published:** 2025-07-03

**Authors:** Han Wang, Di Zuo, Fei Zheng, Yingying Sun, Yuxuan Liu, Hao Yang, Jingfu Wang, Xiaorong Sun

**Affiliations:** ^1^Department of Nuclear Medicine, Shandong Cancer Hospital and Institute, Jinan, China; ^2^Department of Nuclear Medicine, Sichuan Provincial People's Hospital East Sichuan Hospital & Dazhou First People's Hospital, Dazhou, China; ^3^Department of Pediatric Oncology, Shandong Cancer Hospital and Institute, Jinan, China

**Keywords:** neuroblastoma, positron emission tomography/computed tomography (PET/CT), ^18^F-AlF-NOTATATE, restage, chemotherapy

## Abstract

**Purpose:**

To evaluate the value of ^18^F-AlF-NOTATATE PET/CT in restaging high-risk neuroblastoma (NB) after chemotherapy and its advantages over anatomical imaging.

**Materials and methods:**

We retrospectively collected data from high-risk patients with NB who were restaged after chemotherapy using ^18^F-AlF-NOTATATE PET/CT between June 2021 and June 2022. The histopathological, clinical, and radiographic follow-up results were used as reference standards for the final diagnosis. Patient- and lesion-based analyses were performed. The chi-square test was used to compare the efficacy between ^18^F-AlF-NOTATATE PET/CT and anatomical imaging for the detection of residual lesions. One-way ANOVA was used to compare the difference between the maximum standard uptake (SUVmax) values of false-positive and true-positive residual lesions in the surgical subgroups.

**Results:**

Exactly 159 high-risk patients with NB underwent ^18^F-AlF-NOTATATE PET/CT restaging. Exactly 134 patients had 634 residual lesions, and the true-positive rate was 88.5%. Among the residual NB lesions, distant and regional lymph node metastases accounted for 86 and 16%, respectively. In the lesion-based analysis, the sensitivity, specificity, positive predictive value (PPV), negative predictive value (NPV), and accuracy of ^18^F-AlF-NOTATATE PET/CT were significantly higher than those of anatomical imaging. In the patient-based analysis, the specificity, PPV, and accuracy of ^18^F-AlF-NOTATATE PET/CT were also significantly higher. In the surgical subgroup, the SUVmax of false-positive lesions was significantly lower than that of NB.

**Conclusion:**

The efficacy of ^18^F-AlF-NOTATATE PET/CT in restaging high-risk NB after chemotherapy is significantly superior to anatomical imaging. Although, the SUVmax may help identify false-positive lesions, it cannot distinguish benign transformation after NB treatment.

## Introduction

Neuroblastoma (NB) is the most common extracranial malignant solid tumor in children, accounting for 8–10% of all malignant tumors in children, with a mortality rate of up to 15% (1). It typically occurs in children younger than 10 years, with a median age of 18 months at diagnosis. Nearly 60% of patients have already developed metastases at the time of diagnosis, with the most common sites of metastasis being the bone and bone marrow, followed by the lymph nodes (2–4). The treatment plan for high-risk NB includes three phases: Induction Therapy (surgery + chemotherapy), Consolidation Therapy (sequential transplantation + consolidation radiotherapy for the primary site), and Post-Consolidation Therapy (immunotherapy + 13-CRA treatment). The overall survival rate of high-risk patients with NB is less than 50% (1, 4). Therefore, accurate and reliable imaging methods are crucial for determining optimal treatment strategies. However, traditional anatomical imaging techniques, such as ultrasound (US), computed tomography (CT), and magnetic resonance imaging (MRI), have relatively low sensitivity and specificity for the diagnosis and assessment of NB.

NB express norepinephrine transporters that can be labeled with mesoiodobenzylguanidine (MIBG). ^123^I-MIBG imaging has a high sensitivity (88–92%) and specificity (83–92%) for NB and its metastatic foci. Moreover, it has been widely used for the diagnosis of NB, evaluation of the initial treatment, and detection of response to NB treatment (5). However, the accessibility of MIBG imaging in China and its ability to detect small lesions are limited. Compared with MIBG imaging, ^18^F-Fluorodeoxyglucose (^18^F-FDG) PET/CT is less specific for NB and is currently used as a second-line imaging agent, mainly in patients with NB who have negative MIBG uptake and who cannot uptake MIBG during or after treatment (6). The NB surface somatostatin receptors (SSTR) is usually overexpressed in patients with the condition. In recent years, ^68^Ga-labeled somatostatin analog (^68^Ga-SSA) PET/CT has shown potential benefits in the restaging of NB and the assessment of treatment response (7). However, the high cost of ^68^Ge/^68^Ga generators and the short half-life of 68Ga have hindered the application of ^68^Ga-labeled tracers (8). Recently, ^18^F-AlF-NOTA-Octreotide (^18^F-OC) was shown to be a promising tracer for SSTR imaging (9). It has been shown that ^18^F-OC can be an effective alternative to ^68^GA-DOTA-Octreotide (^68^GA-OC) for SSTR PET/CT imaging in patients with Neuroendocrine Tumor (NET) and has better detection efficacy for liver lesions (10). However, the clinical value of ^18^F-OC PET/CT in pediatric patients with NB requires further investigation.

This retrospective study aimed to evaluate the value of ^18^F-AlF-NOTATATE PET/CT in restaging high-risk NB after chemotherapy and compare it with conventional imaging (co-located CT ± contemporaneous liver/brain-enhanced MRI).

## Materials and methods

This retrospective study was approved by the appropriate Ethics Committee, and the patients signed an informed consent form. Data from high-risk patients with NB who underwent ^18^F-AlF-NOTATATE PET/CT for restaging between June 2021 and June 2022 were retrospectively collected. Contemporaneous (within 1 month) liver- and brain-enhanced MR scan results were obtained from the enrolled patients. The inclusion criteria were as follows: (1) pediatric patients (age ≤18 years); (2) pathologically confirmed NB; (3) previous induction, consolidation, or post-consolidation chemotherapy; (4) risk stratification as a high-risk group; and (5) complete clinical and follow-up data. The results of the patient’s biopsy (referred to as biopsy) or reoperation with postoperative pathology, laboratory tests, and clinical and imaging follow-ups (at least 6 months) were used as the gold standard for the final diagnosis.

All images were acquired on a PET/CT scanner (Biographversion; Siemens, Germany). PET tracers were automatically produced with labelled nuclide ^18^F using the accelerator MINI trace from GE, USA, ^18^F-AlF-NOTATATE with ≥95% purity of the putative.

All images were acquired using a PET/CT scanner (Biograph version; Siemens, Germany), which scanned from the top of the skull to the soles of the feet after intravenous administration of ^18^F-AIF-NOTATATE at a dose of 3.3 to 4.44 MBq/kg. Fasting and normal blood glucose levels were not assessed. ^18^F-AIF-NOTATATE PET/CT was performed 2 weeks after chemotherapy and somatostatin analog treatment. A PET scan was performed with 3 min per frame 3D acquisition. Low-dose CT (120 kV) was performed for attenuation correction, and all corrections were applied to the reconstructed images to provide anatomical information. All data were reconstructed using an iterative algorithm (Siemens).

The PET images were reviewed by two experienced nuclear medicine physicians in a blinded manner, and disagreements were discussed and resolved. Increased ^18^F-AlF-NOTATATE PET uptake above the surrounding tissue background, with/without morphological changes, was considered a positive lesion when physiologic uptake and definite benign disease were excluded. A negative diagnosis was made when PET did not show abnormal concentrations. The region of interest was outlined by selecting the site with the highest concentration on the 3D image. The maximum standardized uptake value (SUVmax) of all positive lesions was measured, and the lesions were scored according to the Krenning score (11) (0 = no uptake; 1 = uptake above background but less than liver; 2 = uptake slightly less than or equal to liver; 3 = uptake greater than liver; 4 = uptake greater than spleen). The total number of bone lesions per patient was calculated by PET and CT. Multiple and diffuse uncountable lesions were arbitrarily divided into 10 groups. The nuclear medicine physicians were also asked to characterise the bone lesions (dissolving, sclerotic or mixed) on the basis of visual analysis.

Following the ^18^F-AIF-NOTATATE PET/CT examination, all patients will undergo a one-week follow-up to meticulously monitor and document any adverse reactions associated with this diagnostic procedure. These adverse reactions include fever, edema, pain, nausea, vomiting, diarrhea, and injection site reactions. According to the common terminology criteria for adverse events (CTCAE) issued by the National Cancer Institute (NCI) under the National Institutes of Health (NIH), the above adverse reactions were graded to evaluate their severity.

Statistical analyses were performed using IBM SPSS software (version 26.0). The χ^2^ test was used to compare the efficacy of ^18^F-AlF-NOTATATE PET/CT with that of conventional anatomical imaging for residual lesion detection. One-way ANOVA was also used to compare the difference in SUVmax between false-positive and true-positive residual lesions in the surgical subgroups. The level of statistical significance was set at *p* < 0.05.

## Results

Among 159 children, 88 (55.3%) were boys and 71 (44.6%) were girls. These patients did not experience any adverse reactions caused by the radiotracer ^18^F-AlF-NOTATATE after undergoing PET/CT. Exactly 124 (78%) patients were treated with first-line therapy for high-risk NB, and 35 (22%) experienced recurrence. Of these, 634 positive lesions were found in 134 patients, and 561 (95.9%) were confirmed as true-positive lesions by postoperative pathology and imaging follow-up (*n* = 54, determined by postoperative pathology; *n* = 507, determined by imaging follow-up). The primary tumor sites included the retroperitoneum in 144 patients (88%), the posterior mediastinum in 17 patients (11%), and the neck in two patients (1%). Residual lesions after induction, consolidation, or post-consolidation chemotherapy were mainly located in distant metastases in approximately 86% of the cases, with bone and bone marrow metastases accounting for the highest percentage (60%), followed by regional lymph nodes, non-regional lymph nodes, the brain, the primary site, the liver and lungs, the intra- or paravertebral soft tissues, the pleura and adrenal glands, and the muscles and intraorbital soft tissues in that order. Fifteen of the 25 negative patients underwent chemotherapy after PET imaging. The median follow-up period was 251 days, and eight patients did not experience recurrence. The patient characteristics are detailed in Table 1.

**Table 1:** Patient and clinical characteristics (*n* = 159).

Characteristics	Number(%) of patients or median(range)
Age(y)	
Median age (years)	4(1–17)
Gender	
Male	88(55%)
Female	71(45%)
Primary tumor	
Abdomen	140(88%)
Mediastinum	17(11%)
Neck	2(1%)
INSS	
I	0
II	0
III	14(9%)
IV	145(91%)
Frontline Therapy	124(78%)
Induction Therapy	40(32%)
Consolidation Therapy	73(59%)
Post-Consolidation Therapy	11(9%)
Recrudesce	35(22%)
Response assessment	27(77%)
Monitor	8(23%)
Metastatic site	
T	
Residual primary site	9(6%)
N	
Regional lymph node	26(16%)
M	
Bone and bone marrow	81(51%)
Soft tissues	56(35%)
Non regional lymph node	23(14%)
Brain	14(9%)
Liver	5(3%)
Lung	5(3%)
Intraspinal or paravertebral	3(2%)
Pleural	2(1%)
Adrenal	2(1%)
Leg muscle	1(0.6%)
Intraorbital	1(0.6%)

^18^F-AlF-NOTATATE PET/CT detected NB lesions with a minimum diameter of 1.0 cm for primary residual/recurrent metastasis, 0.2 cm for lymph node metastasis (0.3 cm for regional lymph nodes and 0.2 cm for non-regional lymph nodes), and 0.5 cm for non-lymph node distant metastasis. In addition, of the 460 ^18^F-AlF-NOTATATE PET/CT high uptake bone and bone marrow metastatic lesions, 341 bone and bone marrow metastatic lesions had morphologic and density abnormalities, with predominantly osteolytic changes (48.7%, 166/341). Osteogenic changes accounted for approximately 43.7% of the cases. Residual bone and bone marrow lesions were predominantly found in the limb bones, followed by the vertebrae, pelvis, maxillofacial bones, and skull.

Based on the Krenning score, the degree of uptake in NB lesions reported using ^18^F-AlF-NOTATATE PET/CT was predominantly 1–2 points in 62% of the cases, 3 points in 27%, and 4 points in 9%. Table 2 presents the ^18^F-AlF-NOTATATE PET/CT SUVmax and Krenning scores for the different disease sites.

**Table 2:** The ^18^F-AlF-NOTATATE PET/CT SUVmax and Krenning scores for the different disease sites.

Disease sites	N	Minimum lesion	SUVmax-lesion	Krenning score				
		Diameter	Median(Q1, Q3)	0	1	2	3	4
Residual primary site	10	1.0 cm	9.50(4.48,10.40)	–	–	2	5	3
Regional lymph node	31	0.3 cm	4.96(3.18,8.40)	1	6	9	5	10
Bone and bone marrow	460	–	3.31(2.23,4.83)	–	118	185	130	27
Non regional lymph node	26	0.2 cm	5.34 (2.85,8.60)	2	4	6	6	8
Brain	14	1.3 cm	0.60(0.30,1.40)	3	9	1	1	–
Liver	5	0.7 cm	8.0(6.4,9.30)	1	–	3	1	–
Lung	5	1.0 cm	1	4	1	–	–	–
Other sites	10	0.5 cm	5.83(3.55,6.55)	–	1	3	6	–
Total	561	–	–	11	139	209	154	48

Other sites include intra- or paravertebral soft tissue, pleura, adrenal glands, leg muscles, intraorbital.

In the lesion-based analysis, the sensitivity, specificity, positive predictive value (PPV), negative predictive value (NPV), and accuracy of ^18^F-AlF-NOTATATE PET/CT and co-located CT ± contemporaneous liver/brain-enhanced MRI were 98.0, 80.7, 97.0, 86.6, and 95.7% and 69.5, 35.2, 87.2, 15.3, and 64.9%, respectively. In Table 3, the detection rates of the two imaging modalities for different disease sites are reported based on the final total number of true lesions.

**Table 3:** Detection rates of ^18^F-AlF-NOTATATE PET/CT versus CT/MR per site of disease, according to total number of true lesions at final outcome.

Disease sites	^18^F-AlF-NOTATATE PET/CT		CT/MR		
	Number	%	Number	%	*p*
Residual primary site	10/10	100	9/10	90	–
Regional lymph node	30/31	97	9/31	29	<0.0001
Bone and bone marrow	460/460	100	341/460	74	<0.0001
Non regional lymph node	24/26	92	2/26	8	<0.0001
Brain	11/14	79	14/14	100	–
Liver	4/5	80	5/5	100	–
Lung	1/5	20	2/5	40	–
Other sites	10/10	100	8/10	80	–
Total	550/561	98	390/561	70	<0.0001

*X^2^ test (*p* < 0.05 considered as statistically significant).

The superior sites for residual lesion detection by SSTR PET/CT were regional/non-regional lymph node, bone, and bone marrow metastases. For residual lesion detection at the primary site, there was no difference in diagnostic performance between ^18^F-AlF-NOTATATE PET/CT and conventional imaging; however, ^18^F-AlF-NOTATATE PET had higher sensitivity (100%) and accuracy (100%) than conventional imaging (90 and 99%, respectively). For regional lymph node lesion detection, ^18^F-AlF-NOTATATE PET/CT was significantly superior to conventional imaging, with sensitivities of 97 and 29% (*p* < 0.0001) for ^18^F-AlF-NOTATATE PET/CT and conventional imaging, respectively, and accuracies of 98 and 86% (*p* = 0.0003), respectively. For the detection of distant metastatic lesions, the diagnostic efficacy of ^18^F-AlF-NOTATATE PET/CT for bone and bone marrow metastases and non-regional lymph node metastatic lesions was significantly better than that of conventional imaging.

**Figure 1:**
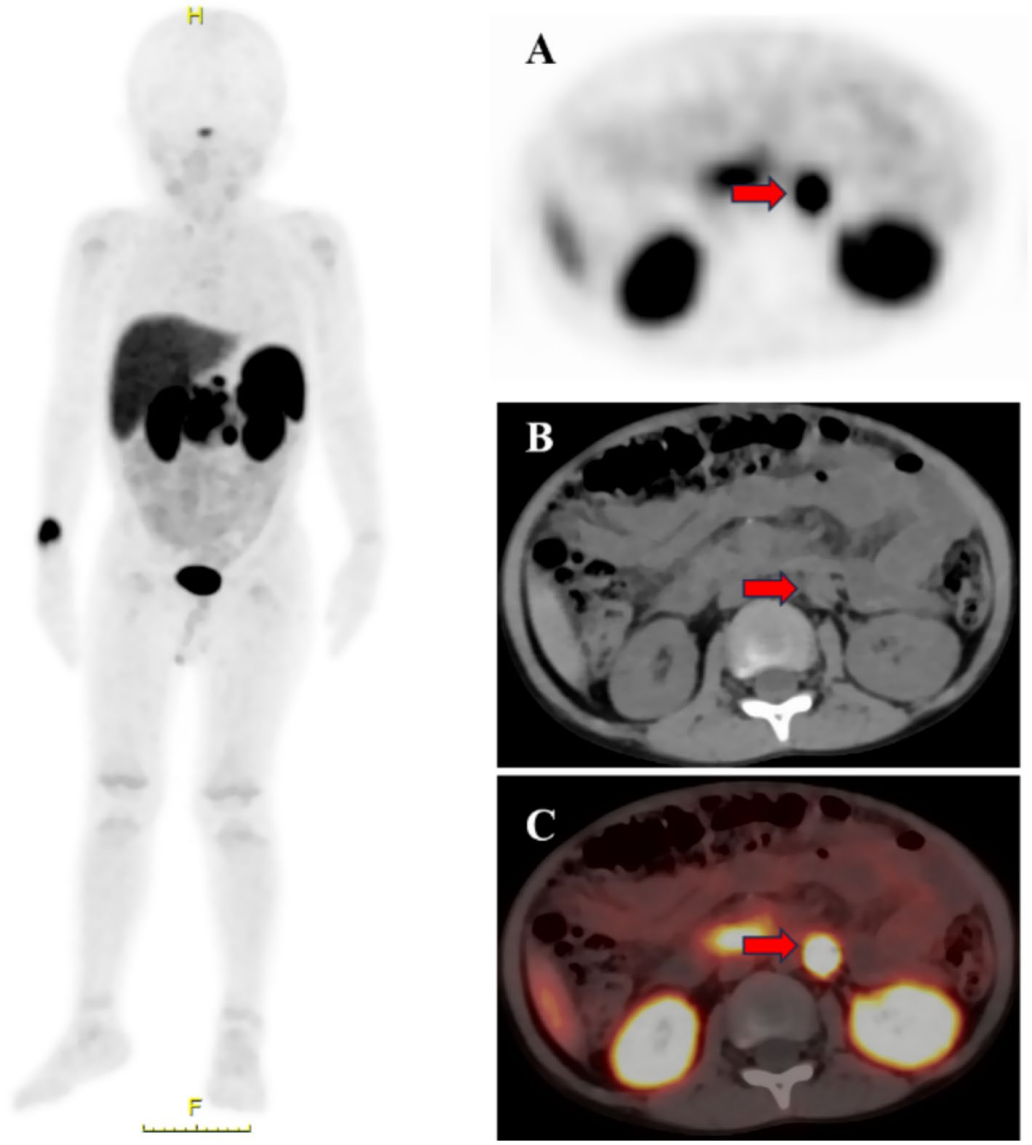
The primary site was located in the retroperitoneum in a child (male, 4 years old), PET showed [**(A)**, red arrow] a rounded hyperintense focus in the left adrenal region; CT on the same machine showed [**(B)**, red arrow] a rounded nodular focus in the left adrenal region with a diameter of approximately 0.9 cm; PET/CT showed [**(C)**, red arrow] a rounded hyperintense focus in the left adrenal region with an SUVmax of 17.9. The final reopening postoperative pathology the diagnosis of ganglion cell neuroma was confirmed.

^18^F-AlF-NOTATATE PET/CT revealed 17 false-positive lesions, including seven cases of ganglion cell neuromas (two cases involving the lymph nodes, two involving the leg muscles, one involving the liver, one involving the left adrenal gland, and one involving the right pleura), six cases of inflammatory lymph nodes, three cases of benign bone lesions, and one case of lung inflammation. Figure 1 illustrates a case of high-uptake ganglion cell neuroma in the left adrenal gland.

**Figure 2:**
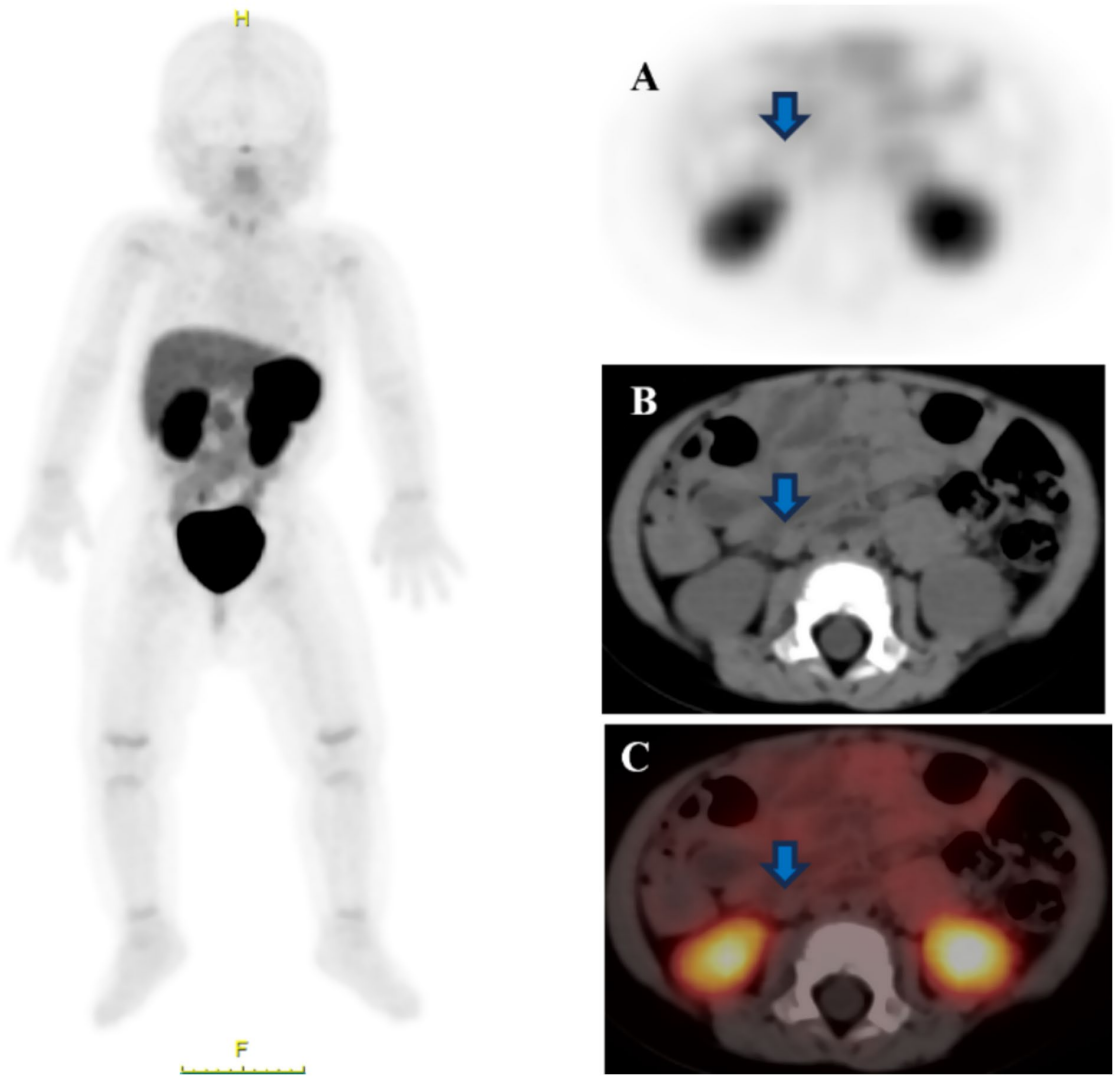
The primary site was located in the retroperitoneum in a child (female, 3 years old), and CT on the same machine showed [**(B)**, blue arrow] a retroperitoneal lymph node with a diameter of approximately 0.5 cm; both PET and PET/CT [**(A)**, blue arrow; **(C)**, blue arrow] did not show any high uptake of nodal foci in the same area. The final reoperation pathology confirmed the diagnosis of metastatic neuroblastoma.

^18^F-AlF-NOTATATE PET/CT revealed 11 false-negative lesions, including four in the lungs, three in the brain, three in the lymph nodes, and one in the liver. Lymph node metastatic lesions in the retroperitoneum are illustrated in Figure 2.

In the patient-based analysis, the sensitivity, specificity, PPV, NPV, and accuracy of ^18^F-AlF-NOTATATE PET/CT and co-located CT ± contemporaneous liver/brain-enhanced MRI were 96.9, 89.4, 93.2, 95.1, and 93.9%, and 96.9, 53.0, 75.6, 92.1, and 69.3%, respectively. Moreover, SSTR PET was significantly more specific in terms of restaging, PPV, NPV, and ACC. Data regarding the diagnostic efficacies of patient-based ^18^F-AlF-NOTATATE PET/CT and conventional imaging for NB are presented in Table 4.

**Table 4:** Diagnostic performance of CT/MRI and ^18^F-AlF-NOTATATE PET/CT per site of disease.

Disease sites	Imaging method	Sen.	Spe.	PPV	NPV	Acc.
Residual primary site	PET/CT	100%	100%	100%	100%	100%
	CT	90%	100%	100%	99%	99%
	*P*	–	–	–	–	–
Regional lymph node	PET/CT	97%	98%	91%	99%	98%
	CT	29%	99%	90%	86%	86%
	*P*	<0.0001	0.43	–	<0.0001	0.0003
Bone and bone marrow	PET/CT	100%	96%	99%	100%	99%
	CT	74%	40%	87%	22%	69%
	*P*	<0.0001	<0.0001	0.84	<0.0001	<0.0001
Non regional lymph node	PET/CT	92%	96%	83%	98%	96%
	CT	8%	99%	67%	85%	84%
	*P*	<0.0001	0.23	–	<0.0001	0.001
Brain	PET/CT	79%	100%	100%	98%	98%
	MR	100%	100%	100%	100%	100%
	*P*	0.22	–	–	0.25	0.257
Liver	PET/CT	80%	99%	80%	99%	99%
	MR	100%	99%	83%	100%	99%
	*P*	–	–	–	–	–
Lung	PET/CT	20%	99%	50%	97%	97%
	CT	40%	99%	83%	100	99%
	*P*	–	–	1	0.14	–
Other site	PET/CT	100%	99%	71%	100%	98%
	CT	80%	99%	72%	99%	97%
	*P*	0.46	–	–	–	0.99
Total	PET/CT	98%	81%	97%	87%	96%
	CT ± MR	70%	35%	87%	15%	65%
	*P*	<0.0001	<0.0001	<0.0001	<0.0001	<0.0001

Other sites, intra- or paravertebral soft tissues, pleura, adrenal glands, leg muscles, and intraorbital metastases; Sen., sensitivity; Spe., specificity; PPV, positive predictive value; NPV, negative predictive value; Acc., accuracy.

**Figure 3:**
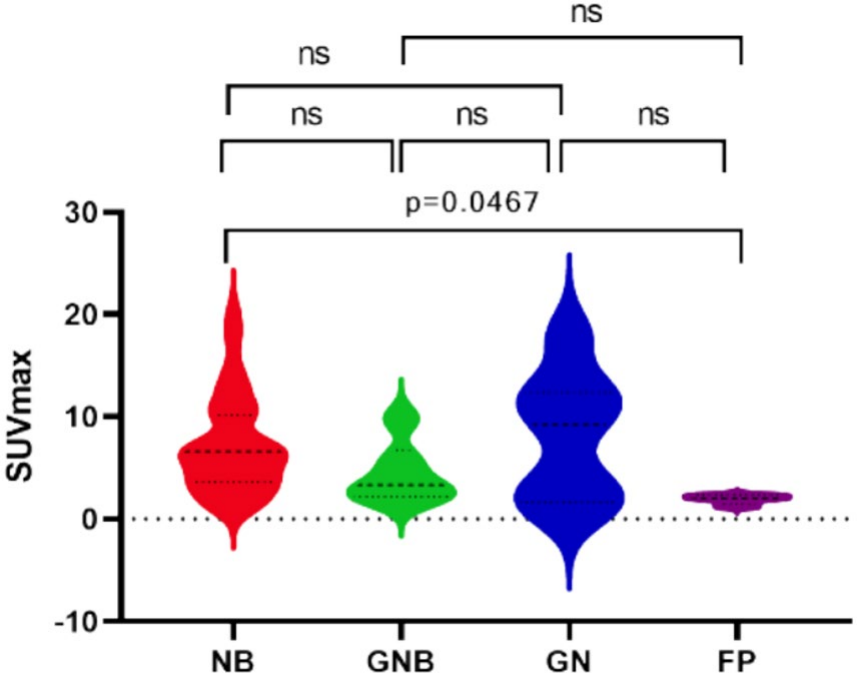
One-way ANOVA ^18^F-AlF-NOTATATE parameters (SUVmax) of NB in different pathologic. NB, Neuroblastoma; GNB, Ganglioneuroblastoma; GN, Ganglioneuroma; FP, False Positive.

Seventy-three lesions were surgically resected, and the pathological findings after reoperation were NB (n = 54, SUVmax = 6.70 (3.67,10.00)), ganglion cell neuroblastoma (GNB, n = 6, SUVmax = 1.46 (2.06,4.44)), ganglion cell neuroma (GN, n = 7, SUVmax = 9.20 (2.72,12.05)), and false-positive lesions (n = 6, SUVmax = 2.01 (1.70,2.23)). One-way ANOVA revealed that the difference in SUVmax among NB, GNB, GN, and the false-positive lesions was statistically significant (*p* = 0.036). Moreover, there was a difference in SUVmax between NB and false-positive lesions. The SUVmax of the false-positive lesions was significantly lower than that of the NB lesions; however, it was not statistically different from the GNB and GN lesions (Figure 3).

## Discussion

In this study, by comparing ^18^F-AlF-NOTATATE PET/CT and conventional imaging (same-computer CT with/without simultaneous enhanced MRI) in all patients, ^18^F-AlF-NOTATATE PET/CT was found to be more sensitive than conventional imaging for detecting residual lesions after chemotherapy in patients with high-risk NB, especially in detecting residual bone, bone marrow, and lymph node occult lesions. The higher sensitivity and specificity of the technique could potentially be an important alternative to ^123^I-MIBG imaging to improve the accuracy of restaging after chemotherapy for patients with high-risk NB and help clinicians revise treatment strategies in a timely manner to improve patient prognosis. In addition, in the surgical subgroup analysis, the SUVmax showed potential for screening NB and false-positive lesions.

In this study, we observed that in patients with high-risk NB who underwent induction, consolidation, or post-consolidation chemotherapy, the residual lesions were predominantly distant metastases, similar to previously reported results (4). The incidence of bone and bone marrow metastases in this study was high (51%), and the metastases were widely distributed, mainly accumulating in the limb bones and spine. In addition, lymph node metastasis was more common, with abdominopelvic and retroperitoneal lymph node metastasis prevalent in NB originating in the retroperitoneum and mediastinal and clavicular lymph node metastasis predominating in NB located in the posterior mediastinum. Smaller lymph node metastases were clearly observed by high uptake on ^18^F-AlF-NOTATATE PET. Further analysis showed that the Krenning score of residual lesions after chemotherapy was predominantly 1–2 on ^18^F-AlF-NOTATATE PET/CT. This suggests that by using ^18^F-AlF-NOTATATE PET/CT to detect high-risk NB post-chemotherapy, residual lesions can be correctly identified, even with a low SUVmax.

Secondly, this study investigated the diagnostic efficacy of ^18^F-AlF-NOTATATE PET/CT in the restaging of high-risk NB after chemotherapy and compared it with the co-located CT ± contemporaneous liver/brain-enhanced MRI. The results revealed that ^18^F-AlF-NOTATATE PET/CT demonstrated higher diagnostic efficacy, particularly in terms of sensitivity, specificity, and accuracy, in the patient-based and lesion-based analyses. This result is similar to that of Rahaf et al., who compared the effects of 68Ga-DOTATATE PET/CT and MRI on the restaging and assessment of treatment response in eight children with NB (7).

In the lesion-based analysis, ^18^F-AlF-NOTATATE PET/CT was found to be more sensitive in detecting the localization of residual lesions in the bone, bone marrow, and occult lymph nodes. This result may be attributed to the size reduction of residual lesions after chemotherapy and the reduction in tumor activity. CT has a low detection rate for diagnosing tiny lesions (<1 cm) in the peritoneum, lymph nodes and bone due to partial volume effect (12, 13). It is well-known that after treatment, PET functional imaging is usually able to detect residual lesions better than CT/MRI (14). The limitations of CT/MRI in the assessment of bone and bone marrow involvement arise mainly from the limitations of its imaging range, as CT or MRI is usually acquired in a dedicated field of view or is confined to axial areas (e.g., abdomen and thorax), whereas PET/CT provides whole-body detection modalities. In addition, CT/MRI is prone to misdiagnosis in the assessment of residual bone and bone marrow lesions after NB chemotherapy, mainly because patients often have irreversible changes in bone mineral density and morphology despite effective control of tumor activity after treatment. In the detection of lymph node lesions, CT is diagnosed by measuring the short meridian of the lymph nodes (usually, a short meridian of more than 1.5 cm is considered a metastatic lymph node), whereas PET is determined by the uptake of ^18^F-AlF-NOTATATE. Thus, PET/CT demonstrated greater sensitivity for detecting lymph node metastasis. It is worth noting that although ^18^F-AlF-NOTATATE PET/CT performed well in most aspects, its sensitivity was slightly lower than that of conventional imaging techniques in the detection of residual lesions in the liver, which may be due to the uptake of ^18^F-AlF-NOTATATE by normal cells in the liver, resulting in lower target samples of liver lesions, which may mask some of the small foci. The same has been found in previous studies (15). ^18^F-AlF-NOTATATE PET/CT was slightly less accurate than MRI in detecting residual brain lesions, with three missed cases. This finding suggests that a combination of imaging techniques should be used for the diagnosis of brain and liver metastases to improve diagnostic accuracy.

In this study, although ^18^F-AlF-NOTATATE PET/CT showed significant advantages in detecting residual lesions of NB, 11 cases of residual lesions were not detected. Based on previous research (16), the possible reasons for these non-detection cases were explored in depth and are now attributed to the following: (1) The residual lesions had a low level of SSTR expression, which led to a reduction in the contrast of the lesions on PET/CT imaging and posed a diagnostic difficulty. (2) Influence of the high radiological background region: If the residual lesion was located in a region with a high radiological background, such as the liver, spleen, or bladder, the uptake of ^18^F-AlF-NOTATATE by normal cells or tissues in these regions may have been higher, thus masking ^18^F-AlF-NOTATATE uptake in the region of the residual lesion, which made it difficult for the lesion to be detected. (3) Concealment and size limitation of residual lesions: When the size of the residual lesions was too small, in a concealed location, or in proximity to the primary foci, these lesions may have been difficult to detect accurately owing to the partial volume effect of PET/CT and the limitation of spatial resolution. Additionally, a low degree of ^18^F-AlF-NOTATATE uptake was observed at sites of inflammation/infection, post-radiotherapy alterations, and osteoblastic activity.

In the patient-based analyses, ^18^F-AlF-NOTATATE PET/CT showed improved specificity, PPV, and ACC for restaging high-risk NB after chemotherapy. This may be attributed to the accurate identification of residual bone and bone marrow lesions using PET/CT after chemotherapy. These findings provide a more accurate diagnostic basis for restaging high-risk patients with NB.

Finally, in the one-way ANOVA, the SUVmax demonstrated some potential in screening NB and false-positive lesions but was unable to effectively differentiate between GN transformations after NB treatment. Post-treatment GN transformation of NB is a complex biological phenomenon that often occurs during post-treatment follow-up. This transformation may lead to changes in the metabolic properties of the primary lesion, rendering the SUVmax unable to accurately reflect its pathophysiological status. Future studies should explore the use of SUVmax in combination with other clinical indicators to improve the diagnostic accuracy of GN transformation after NB treatment. Simultaneously, researchers can explore new imaging parameters to assess changes in metabolic activity in NB and provide clinicians with more accurate diagnostic and therapeutic decision support.

This study had some limitations. First, the sample size was relatively small, which may have some impact on the stability and reliability of the results. Second, owing to the retrospective nature of the study, some patients were scanned using various CT, MRI, and PET protocols with different scanners, resulting in inconsistent imaging parameters. Finally, some NB cases were confirmed histopathologically via tumor biopsies and radiographic follow-ups. However, sampling and radiographic features may not be representative owing to intratumoral expression heterogeneity.

## Conclusion

Our results demonstrate that ^18^F-AlF-NOTATATE PET/CT has significantly better diagnostic efficacy than conventional imaging in the restaging of high-risk NB after chemotherapy, especially in the detection of occult lesions, which may help improve the accuracy of NB restaging, thus providing physicians with more accurate diagnostic information and guiding more effective treatment options. Despite the excellent performance of ^18^F-AlF-NOTATATE PET/CT for the detection of occult lesions, it is important to note the limitations of this technique. Nodular cell neuroma and inflammatory lymph nodes may be misdiagnosed, and liver and brain metastases may be underdiagnosed. Future studies should explore how to maximize the use of ^18^F-AlF-NOTATATE PET/CT in combination with other imaging methods to achieve a more comprehensive and accurate neuroblastoma diagnosis.

## Data Availability

The original contributions presented in the study are included in the article/supplementary material, further inquiries can be directed to the corresponding authors.
